# The Role of Temporal Acoustic Exaggeration in High Variability Phonetic Training: A Behavioral and ERP Study

**DOI:** 10.3389/fpsyg.2019.01178

**Published:** 2019-05-24

**Authors:** Bing Cheng, Xiaojuan Zhang, Siying Fan, Yang Zhang

**Affiliations:** ^1^English Department & Language and Cognitive Neuroscience Lab, School of Foreign Studies, Xi’an Jiaotong University, Xi’an, China; ^2^Department of Speech-Language-Hearing Sciences, Center for Neurobehavioral Development, University of Minnesota, Minneapolis, MN, United States

**Keywords:** acoustic exaggeration, HVPT, categorical perception, mismatch negativity, second language learning

## Abstract

High variability phonetic training (HVPT) has been found to be effective in helping adult learners acquire non-native phonetic contrasts. The present study investigated the role of temporal acoustic exaggeration by comparing the canonical HVPT paradigm without involving acoustic exaggeration with a modified adaptive HVPT paradigm that integrated key temporal exaggerations in infant-directed speech (IDS). Sixty native Chinese adults participated in the training of the English /i/ and /i/ vowel contrast and were randomly assigned to three subject groups. Twenty were trained with the typical HVPT paradigm (the HVPT group), twenty were trained under the modified adaptive approach with acoustic exaggeration (the HVPT-E group), and twenty were in the control group. Behavioral tasks for the pre- and post- tests used natural word identification, synthetic stimuli identification, and synthetic stimuli discrimination. Mismatch negativity (MMN) responses from the HVPT-E group were also obtained to assess the training effects in within- and across- category discrimination without requiring focused attention. Like previous studies, significant generalization effects to new talkers were found in both the HVPT group and the HVPT-E group. The HVPT-E group, by contrast, showed greater improvement as reflected in larger progress in natural word identification performance. Furthermore, the HVPT-E group exhibited more native-like categorical perception based on spectral cues after training, together with corresponding training-induced changes in the MMN responses to within- and across- category differences. These data provide the initial evidence supporting the important role of temporal acoustic exaggeration with adaptive training in facilitating phonetic learning and promoting brain plasticity at the perceptual and pre-attentive neural levels.

## Introduction

Learning non-native speech sounds can be a particularly challenging task. For example, the distinction of the English vowel contrast /i/-/i/ is exceptionally difficult for many native Chinese speakers who learn English as a second language (L2) (Flege et al., 1997; Wang, 1997; Wang and Heuven, 2006; Cheng and Zhang, 2013; Liu et al., 2014; Huang et al., 2018). The literature has well documented that high variability phonetic training (HVPT) is effective on improving non-native speech perception. This improvement can generalize to new contexts and talkers, and transfer to production (Logan et al., 1991; Lively et al., 1993; Strange, 1995; Bradlow and Pisoni, 1997; Wang et al., 1999; Iverson et al., 2005; Sadakata and Mcqueen, 2013). However, not all L2 learners appear to benefit from HVPT; the effectiveness is limited by learners’ perceptual abilities, first language (L1) background, and the nature of speech categories to be learned (Iverson and Evans, 2009; Perrachione et al., 2011; Sadakata and Mcqueen, 2014; Grenon et al., 2019). Although earlier studies showed the efficacy of HVPT only with the identification training protocol, a recent study demonstrated the feasibility of both identification and discrimination training with similar improvements (Shinohara and Iverson, 2018). Some studies further indicated the benefits of combining systematic temporal/spectral exaggeration with adaptive training in the HVPT paradigm (Zhang et al., 2000, 2009; Grenon et al., 2019) while achieving limited success in overcoming the native language interference. But the effects of this modified and integrated HVPT approach have not been directly compared with those without introducing the temporal acoustic exaggeration.

The present investigation aimed to compare the two HVPT protocols in training adult Chinese speakers to learn the English vowel contrast /i/-/i/. We included two training groups and one control group to test the efficacy and generalizability of the integrated training approach combining the features of high variability and temporal acoustic exaggeration with adaptive learning motivated by infant-directed speech (IDS), which has been previously shown to promote adult L2 perceptual learning (Iverson et al., 2003; Zhang et al., 2009). We also supplemented the behavioral results with electrophysiological data to examine the training-related neural plasticity in acquiring L2 speech categories, which can provide insights into the nature of underlying mechanisms of non-native phonetic learning in adulthood at both attentive and pre-attentive levels.

### Non-native Phonetic Training Methods

Developmental speech perception studies have shown a strong native language neural commitment process early in life that facilitates L1 learning and constrains the infant’s ability to perceive non-native speech contrasts (Kuhl, 2000). Despite the limitations from L1 interference, adult learners can improve their L2 perception and production at least for some non-native speech sounds, indicating that the adult brain has more neural plasticity than was ever believed (Pisoni et al., 1982; Strange and Dittmann, 1984; Jamieson and Morosan, 1986; Logan et al., 1991; Lively et al., 1993, 1994; Bradlow and Pisoni, 1997; Wang et al., 1999). Based on the assumption that adult perceptual mechanisms can be retuned, auditory training studies have utilized a variety of short-term intensive laboratory training methods to improve non-native speech perception, which can result in robust learning in terms of generalization to new phonetic contexts and new talkers (Francis et al., 2000; McCandliss et al., 2002; Pruitt et al., 2006). However, it seems that effective generalization may depend on whether learners encounter sufficiently variable stimuli during training. For example, Strange and Dittmann (1984) trained native Japanese speakers to learn the English contrast /r/-/l/ using the stimuli along a synthetic continuum of *rock*-*lock*. The results showed that despite improvements in discrimination and identification after training on the trained synthetic stimuli and on a novel synthetic continuum, the training effects did not appear to transfer to naturally-produced minimal pairs. Later on, Logan et al. (1991) also trained native Japanese speakers to perceive the English /r/-/l/ contrast, and they introduced highly variable stimuli including multiple natural minimal pairs produced by multiple talkers (i.e., HVPT). The results revealed improvements in both trained and untrained stimuli. Lively et al. (1993) replicated the generalization effect of HVPT. When using the same HVPT paradigm with the training stimuli produced by a single talker in a follow-up experiment, they found improvements for the trained talker but not for a new talker, which indicates an important role for high talker variability in phonetic training.

Since those early attempts, the effectiveness of HVPT has been reported on a variety of phonetic contrasts with some studies showing benefits of long-term retention and transfer of learning from perception to production (Lively et al., 1994; Bradlow and Pisoni, 1997; Bradlow et al., 1999; Sakai and Moorman, 2018). However, more recent phonetic training research has revealed some mixed results of HVPT training effects. On the one hand, a great number of studies showed that training with highly variable stimuli was more effective for L2 phonetic learning than low-variability training (Wang et al., 1999; Hardison, 2003; Brosseau-Lapré et al., 2013; Sadakata and Mcqueen, 2013; Zhang et al., 2018). On the other hand, other studies demonstrated that variability may hinder learning difficult L2 contrasts in some circumstances (Wayland and Guion, 2004; Wade et al., 2007; Chang and Bowles, 2015; Antoniou and Wong, 2016; Giannakopoulou et al., 2017). For example, Wade et al. (2007) showed that variability diminishes learning effects on highly confusable English vowels, relative to less confusable ones. Additionally, variability appears to diminish learning effects for novice learners (Wayland and Guion, 2004; Chang and Bowles, 2015), but it still is possible for novice learners to benefit from variability if they have strong perceptual abilities (Perrachione et al., 2011; Sadakata and Mcqueen, 2014; Antoniou and Wong, 2015). These results suggest that the effectiveness of HVPT partly depends on the learner characteristics and the nature of the to-be-learned categories.

One notable aspect in the recent HVPT studies was the efforts to optimize improvements by combining different training methods that are considered to have a positive influence on the underlying processes for phonetic perception. Given that highly variable input may initially present a challenge for the acquisition of difficult L2 sounds, the present study followed up our pilot work (Zhang and Cheng, 2011; Cheng and Zhang, 2013) to incorporate some key exaggerated characteristics of infant-directed speech into the HVPT paradigm. For the purpose of the current study, we chose to test training effects of the two training paradigms (HVPT without acoustic exaggeration versus the modified HVPT-E with exaggeration) on the perception of English /i/ and /i/ by Chinese adults. IDS, also referred to as “motherese,” is the exaggerated speech style used to address infants, with slower speaking rate, simplified language, exaggerated formant structure, frequent repetitions, and exaggerated prosody, which is presumed to help infants form speech categories (Fernald and Kuhl, 1987; Maye et al., 2002; Werker et al., 2007). However, not all aspects of IDS can facilitate speech learning or are intended to promote speech learning (McMurray et al., 2013). For instance, exaggerations in pitch range and contour do not necessarily facilitate phonetic categorization (Trainor and Desjardins, 2002; Kitamura and Burnham, 2003). Nevertheless, some studies have found that when the training input stimuli were systematically manipulated with temporal and spectral exaggeration, adult L2 perceptual learning could be greatly enhanced (Zhang et al., 2000, 2009; Iverson et al., 2005). The critical notion here is that by exaggerating acoustic differences between stimuli, researchers can make the contrast more discriminable, which can be incorporated in a scaffolding structure for step-by-step adaptive training based on the individual learners’ perceptual ability to facilitate phonetic training (Zhang and Cheng, 2011). For example, Zhang et al. (2009) used a computer-assisted training program featuring acoustic exaggeration and multi-talker variability to train Japanese adults who had limited English exposure to learn the English /r/ and /l/ categories. The results showed that the trainees obtained significant improvement in identification with generalization to untrained synthetic and natural stimuli. As Iverson et al. (2005) and Zhang et al. (2009) did not include a training group without the use of acoustic exaggeration for comparison, the tentative claims about the important role of acoustic exaggeration in the HVPT paradigm have not been directly verified. The present study represents our first attempt to fulfill this gap to assess the contribution of acoustic exaggeration to Chinese adult learners’ perception of the difficult English /i/-/i/ contrast by comparing training effects of the canonical HVPT paradigm with naturally-produced input (i.e., input produced in multiple contexts by multiple speakers) and the modified HVPT paradigm with acoustically-exaggerated input delivered in an adaptive fashion.

### Behavioral Measures of Training Effects

Researchers argue that exposure to high variability input helps learners ignore phonetically irrelevant information and retain long-term memory representations of relevant phonetic features, thus facilitating generalization from a trained set to a novel set (Deng et al., 2018). Although considerable training research has demonstrated training-induced improvement of accuracy in identification and discrimination performance, the training-induced improvements do not necessarily indicate that the leaners have formed more robust representations of L2 speech categories. For a stringent test of speech identification and discrimination performance and the effects of transfer of learning on retuning category representations, it is important to assess the learners’ categorical perception (CP) in pre- and post-training tests with a synthetic speech continuum that was not used in the training protocol (Zhang et al., 2009; Zhang and Cheng, 2011).

Categorical perception is the phenomenon in which the categories possessed by an organism influences the organism’s perception (Liberman et al., 1957; Eimas et al., 1971; Kuhl and Miller, 1978; Steinschneider et al., 1995; Zhang et al., 2005; Kuhl et al., 2006; Goldstone and Hendrickson, 2010). As such, perception is “warped” in the way that differences between objects belonging to different categories are amplified, while differences between objects falling into the same category are deemphasized. Thus it is by CP that the perceptual system transforms relatively linear sensory signals into non-linear internal representations. Although there appears to be an innate basis for CP, the boundaries along acoustic continuua between the speech categories can be modified or even lost as a consequence of learning (Roberson et al., 2000). There is also strong evidence demonstrating that CP can be induced by learning alone (Goldstone, 1994; Livingston et al., 1998). Under a spatial metaphor with axes defined by values on relevant dimensions (e.g., Nosofsky, 1986), the perceptual space may undergo a “warping” process in categorization training by moving members of the same category closer together and thus rendering them to be less discriminable. Therefore, examining CP of the target L2 speech sounds with a synthetic continuum in pre- and post- tests can provide feasible fine-grained measures to assess the training effects, which would be a more direct index of learning-induced changes in category representations than simple performance improvement in identifying the naturally produced speech stimuli.

The prototypical pattern of CP is characterized by a phonetic boundary effect, which shows a sudden membership shift between two categories in the identification function and a distinct peak at the category boundry in the discrimination function. Thus CP is operationalized as poor within-category discrimination and significantly better across-category discrimination, despite the fact that the physical differences are equal (Liberman et al., 1957). Early studies reported the prototypical pattern of CP for consonants with enhanced discrimination for across-category pairs of stimuli and diminished discrimination for within-category paris of stimuli in the speech continuum, and some failed to demonstrate categorical effects for vowels (e.g., Liberman et al., 1957; Fry et al., 1962). More recent research, however, was able to show that categorical-like effects are not completely absent for vowels, but the pattern tends to be weaker for vowels than that of consonants (Minagawa Kawai et al., 2005; Altmann et al., 2014). Since learning experience fundamentally shapes the way the brain represents and processes the category structures of speech sounds (Livingston et al., 1998), it is predicted that successful L2 phonetic learning would ideally lead to more robust categorical representations and native-like CP behavior. As a consequence, one would expect to see the sudden shift of category memberships in the identification function as well as enhanced sensitivity at the category boundary without raising within-category sensitivity in the discrimination function.

### Neurophysiological Measures of Training Effects

In addition to behavioral measures, the current study aimed to assess training-induced brain plasticity by measuring the mismatch negativity (MMN) to investigate neural mechanisms underlying non-native speech learning. Previous research showed that training can induce significant changes in neurophysiological responses, suggesting that the adult brain is not strictly bound by the native language neural commitment early in life and that memory traces can be developed in order to encode new phonetic representations (Tremblay et al., 1997; Zhang and Wang, 2007; Zhang et al., 2009; Deng et al., 2018). In this regard, the MMN response, identified as a component of event-related potentials (ERPs), is a valuable tool for investigating speech perception and the formation of memory traces for newly learned speech categories at the pre-attention level. The MMN is typically elicited within the oddball paradigm when a change occurs in a stimulus stream with the repetitive standard stimulus replaced by infrequent deviant stimulus differing slightly in various parameters from standard stimulus. No focused attention on detecting the stimulus change is required. The MMN quantification requires subtracting the ERP of the frequent standard stimulus from that of the rare deviant stimulus with a negative peak occurring in the time window of 100 to 250 ms following the onset of detectable acoustic change in the deviant stimulus (for a review, see Näätänen, 2001). Previous research has shown varying (i.e., from non-significant to significant) degrees of association between MMN responses and behavioral discrimination performance under different experimental conditions (e.g., Näätänen et al., 1993; Tiitinen et al., 1994; Kujala et al., 2001; Novitski et al., 2004; Chen and Sussman, 2013; Yu et al., 2017). There were also different findings regarding whether the MMN can reflect the category boundary effect in categorical perception (e.g., Ylinen et al., 2006; Xi et al., 2010). For instance, Ylinen et al. (2006) suggested that the status of the deviant relative to the phoneme boundary did not affect the MMN amplitude; that is, the MMN responses to within- and across- category differences did not differ regardless of whether the listeners demonstrated CP for the target sounds or not. On the contrary, in studying the neurophysiological correlates of categorical perception of Chinese lexical tones, Xi et al. (2010) found that the across-category deviants elicited larger MMN than that of the within-category deviants in native speakers of Chinese, and this phenomenon was not observed in the non-native speakers. Despite the controversies, speech research has indeed documented that the MMN can reflect learning-induced changes with enhanced MMN amplitude and reduced MMN latency (Menning et al., 2002; Kujala and Näätänen, 2010).

There are at least two advantages of using the MMN in a speech training study. First, the MMN is measured without requiring the participant’s attention, which allows a more objective test of the perceptual changes at the pre-attentive level. Second, the MMN can index not only the final behavioral outcome, but also capture the significant progress on the way toward a successful outcome, since the MMN may emerge prior to behavioral manifestation of a significant change (Näätänen et al., 1982; Näätänen and Gaillard, 1983; Alho and Sinervo, 1997). If the MMN reflects training effects and corresponds to behavioral measures, it can be predicted that changes in the MMN amplitudes elicited by across-category differences from the pre-test to the post-test should be significantly larger, while the MMN elicited by within-category differences should not change significantly. This learning process may also be accompanied with shorter MMN latency for the across-category discrimination but not for the within-category discrimination.

### Current Study

The current study adds to the speech training literature by examining the role of temporal acoustic exaggeration in the HVPT paradigm to train Chinese adults to learn the English /i/-/i/ vowel contrast. The English tense and lax front unrounded vowels (/i/-/i/ contrast) have been shown to be exceptionally difficult for Chinese speakers. Acoustically, English /i/ has a lower first formant (F1) and higher second formant (F2) than /i/, and /i/ is also typically longer than /i/ in duration. Unlike English, Mandarin Chinese has only one single category of /i/ which is likewise characterized by its low F1 and high F2 (Hillenbrand et al., 1995). According to the Perceptual Assimilation Model (Best, 1995), the fact that the two categories of English vowels /i/-/i/ assimilate into the single Mandarin Chinese /i/ category predicts the difficulty for Chinese adults to learn the contrast. Empirical research evidenced this difficulty. Native English speakers can use both duration and formant frequency cues for distinguishing the two sounds (Mermelstein, 1978; Whalen, 1989; Grenon et al., 2019), and they predominantly rely on the spectral cues (Mermelstein, 1978; Hillenbrand et al., 2000). On the contrary, at least at the initial learning stage, English-as-a-second-language (ESL) learners rely dominantly on duration cues rather than spectral cues in both perception and production (Escudero and Boersma, 2004; Morrison, 2005; Wang and Heuven, 2006; Yang, 2011; Liu et al., 2014).

The HVPT approach with acoustic modification has been found to be effective in changing L2 cue weighting of non-native vowel perception. For example, Ylinen et al. (2010) used the HVPT technique with modified acoustic stimuli to equate durations between vowels to train adult Finnish native speakers who relied more on duration to identify the English /i/-/i/ contrast before training, thus forcing the listeners to use spectral cues. Results showed significant improvement in identifying both natural and duration-modified stimuli. Giannakopoulou et al. (2013) replicated the effects with training native Greek ESL learners to distinguish the same contrast. However, previous research has highlighted the limiting role of the L1 background, indicating the necessity to test the efficacy of HVPT for L2 learners with different L1s (Iverson and Evans, 2009; Grenon et al., 2019). In addition, previous studies have established the positive effects of talker variability in facilitating second language phonetic learning (e.g., Logan et al., 1991; Hardison, 2003; Zhang et al., 2009). The present study continued this line of work with an aim to investigate the efficacy of the modified HVPT paradigm combined with temporal acoustic exaggeration to enhance Chinese L2 learners’ reliance on spectral cues of the /i/-/i/ contrast as measured by behavioral CP effects and MMN responses on the duration-equated stimuli. In particular, we were interested in examining differences in three measurable outcomes in pre- and post- tests, i.e., (1) whether the two training paradigms would show generalization to novel words and new talkers, (2) whether the learner would develop more native-like categorical perception of the /i/-/i/ contrast based on spectral cues, and (3) whether the training paradigms would produce the predicted MMN changes for non-native phonetic learning.

To this end, we embedded the phonetic learning task in word stimuli, in which training requires identifying minimal pair contrasts that are synchronized with visual cues of the speakers’ articulatory motion in video clips on screen (Zhang and Cheng, 2011). We compared training effects of HVPT-E with acoustically-exaggerated input versus HVPT without acoustically-exaggerated input, with overall exposure trials matched across conditions. Our measures of training effects in the pre- and post- tests were threefold: (1) identification of the target vowels in naturally spoken words to measure training and generalization effects; (2) identification and discrimination of synthetic stimuli for categorical perception tests based on spectral cues alone to measure changes in perceptual sensitivity for across- and within- category discrimination; (3) the MMN responses that correspond to the training-induced changes for across- and within- category discrimination at the pre-attentive level.

## Materials and Methods

### Participants

Sixty native Chinese speakers at Xi’an Jiaotong University participated in this study. Participants were volunteers aged between 18 and 36 years old. They were recruited for participation following the approval and guideline of Institutional Review Board for Biomedical Research at Xi’an Jiaotong University, China. Written informed consent was obtained from each participant with hourly monetary compensation for their participation. The participants were all right-handed and had no history of speech, language or hearing problems or disorders. All of them had studied English for at least 6 years prior to attending college and were taking English courses at the university. No one had the experience of living in an English-speaking country or community for over a month. Participants were randomly assigned to three subject groups, 20 in the HVPT group, 20 in the HVPT-E group, and 20 in a control group that did not receive training.

### Stimuli

In both HVPT and HVPT-E paradigms, 40 different tokens (20 minimal pairs) were trained. A total of 840 trials were included, with 120 trials in each session (7 sessions). In the canonical HVPT paradigm, training stimuli were naturally spoken English words containing the target phonemes of /i/ and /i/. Six native American English speakers (3 males and 3 females) participated as talkers for the training stimuli. Digital video (with audio track) recordings of four native speakers (2 females, 2 males) were used as visual cues in the training program, and the other two (audio track only) were adopted in the progress-monitoring quizzes following each training session. In the modified paradigm (HVPT-E), the natural productions were further synthesized in Praat to be acoustically modified with four levels of temporal exaggeration: 300, 208, 144 and 100% (with no exaggeration). The video frames of each word token in the training program were edited in Final Cut Studio to match the duration at the four levels.

The stimuli adopted in the pre- and post- test included natural word stimuli and synthetic target phoneme stimuli. The natural word stimuli were used in the natural word identification test, recorded by four native American English speakers, two males and two females, all of whom were new to the trainees. Altogether, 160 natural word stimuli were used (20 words × 4 talkers × 2 times). Among the twenty words, 10 words (5 minimal pairs) were selected from the training words and other 10 words (5 minimal pairs) were untrained words.

Synthesized phoneme stimuli were an eleven-step continuum between /i/ and /i/, previously used in the identification and discrimination tests as well as the electrophysiological test to examine categorical perception in native speakers and non-native speakers of English (Cheng and Zhang, 2013). The /i/ and /i/ sounds were first recorded in the “h_d” context in a word list read by a male native English speaker at 44.1 kHz sampling rate and then were digitally processed (overlap-add method) to have an equal duration of 170 ms and fade-in and fade-out time of 10 ms using Sound Forge (SoundForge9, Sony Corporation, Japan). For the /i/ sound, the F1 and F2 frequencies are 355 and 2346 Hz, respectively. For the /i/ sound, F1 and F2 were 435 and 2006 Hz. These two stimuli were then employed as the two endpoints to create the eleven-step continuum using a morphing technique in the STRAIGHT package (Kawahara et al., 1999) on the MATLAB platform (Mathworks Corporation, United States). The stimuli on the continuum were normalized to have equal average RMS (root mean square) intensity.

### Procedures

#### Pre/post-tests

The sixty participants were randomly assigned to three groups, two training groups with 20 in the HVPT group and 20 in the HVPT-E group and one control group with 20 participants. Identical tests were conducted one week before and one week after training. To verify that participants’ perceptual performance of the /i/-/i/ contrast in three groups were not significantly different before training, we conducted one-way ANOVA for the behavioral data in the pre-test. No significant differences between three groups were found in any of the natural word identification [*F*(2,57) = 0.882, *p* = 0.419, η^2^_p_ = 0.030 for the overall natural word identification], synthetic phoneme identification [*F*(2,57) = 0.494, *p* = 0.613, η^2^_p_ = 0.017 for the boundary slope; *F*(2,57) = 0.626, *p* = 0.538, η^2^_p_ = 0.021 for the boundary location; *F*(2,57) = 0.286, *p* = 0.752, η^2^_p_ = 0.010 for the boundary width] and discrimination [*F*(2,57) = 0.027, *p* = 0.973, η^2^_p_ = 0.001 for the across-category pair discrimination; *F*(2,57) = 2.192, *p* = 0.121, η^2^_p_ = 0.071 for the within-category pair discrimination] tests. The MMN data were additionally collected from the HVPT-E group before and after training.

The EEG recording for the HVPT-E group was administrated in an electronically and acoustically shielded room. Continuous EEG data were recorded (sampling rate = 500 Hz; bandwidth = 0.3–30 Hz) using the Net Station System with Net 400 amplifier and a 64-channel Geodesic Sensor Net cap (EGI Inc., United States). All the 64 electrodes had impedances below 5 kΩ during recording. The default setting in the EGI system was used with the vertex electrode Cz as the reference. The participants were instructed to ignore the presented sounds of /i/-/i/ continuum while watching a self-selected muted movie on the 20-inch TV monitor placed three meters away from the participant. The stimuli were 3, 7, and 11 from the 11-step synthesized speech continuum. Those stimuli were chosen based on our previous cross-language pilot study (Cheng and Zhang, 2013). In Cheng and Zhang (2013), Step 3 was 100% identified by 10 native speakers of American English as the phoneme /i/ while Step 7 and Step 11 were identified as the phoneme /i/ with accuracy rates of 96.5 and 100%, respectively. Stimulus presentation followed the Double Oddball Paradigm in Xi et al. (2010), which was implemented in Eprime 2.0 (Psychology Software Tools Inc., United States). In this paradigm, an MMN response can be elicited when the repeated presentation of the standard stimulus (Step 7) is interrupted by either deviant stimulus (Step 3 or Step 11). An across-category stimulus pair (Steps 3 and 7) and a within-category stimulus pair (Step 7 and 11) were chosen as two contrasts which occurred pseudo-randomly; that is, the deviant stimuli were designed not to be presented consecutively. The standard stimulus occurred at the frequency of 80% (960 trials) of all trials and the two deviant stimuli occurred each at 10% (120 trials for each deviant). The inter-stimulus interval ranged from 700 to 800 ms. Sound stimuli were presented binaurally at 75 dB SPL via Etymotic Research ER-1 Insert Earphones. The EEG recording session lasted approximately 30 min (excluding the preparation time).

In order to test training and generalization effects to new talkers and new phonetic contexts (new minimal pair words) in which the target phonemes occur, the natural word identification task used 10 trained words and 10 untrained words, all produced by four new native American English speakers. Each word was presented eight times. In the test, participants were asked to judge whether the word they heard included /i/ or /i/ by clicking the icons of the two phonemes on the screen which were represented in International Phonetic Alphabet (IPA). All participants had learned these IPA symbols as part of their English curriculum since middle school. The experimenter verified that each participant recognized the /i/ and /i/ symbols correctly in association with a target minimal pair “beat” vs. “bit” prior to training.

Both identification and discrimination tasks were used for CP tests with the synthetic stimuli. The identification test required participants to label vowel stimuli. Each stimulus (the eleven steps) from the continuum was randomly presented 20 times. The discrimination test required participants to judge whether pairs of presented stimuli were the same or different. Two different sound pairs (across-category pair: Step 3 and Step 7; within-category pair: Step 7 and Step 11) were presented in random order at 10 times, respectively. For each of the two sound pairs, there were four types in the form of AB, BA, AA, and BB, with AB and BA representing different phonemes in the reverse order and AA and BB representing foil trials with the same phoneme.

#### Training Procedures

As described earlier, each trainee was randomly assigned to one of the two training groups: the HVPT group and the HVPT-E group. The only difference in the two training paradigms was the training input with or without acoustic exaggeration, with all other variables matched across conditions. Both training protocols included 7 sessions (Table 1). It took the HVPT-E group approximately 60 to 90 min to complete training (including the quizzes in between training sessions), and the HVPT group 50 to 80 min, depending on each participant’s pace and response speed.

**TABLE 1:** Talker numbers and stimuli in each session of the training program.

**Session**	**Talker Numbers**	**Stimuli**	
		**HVPT-E**	**HVPT**
Session 1	1	exaggerated 300%	natural
Session 2	2	exaggerated 300%	natural
Session 3	3	exaggerated 300%	natural
Session 4	4	exaggerated 300%	natural
Session 5	4	exaggerated 208%	natural
Session 6	4	exaggerated 144%	natural
Session 7	4	No exaggeration 100% (natural)	natural

The participants in the two training groups were asked to complete the training at their own pace in a sound-treated booth. By clicking either one of the target phoneme icons on the screen, the trainee saw a talker utter a word containing the clicked phoneme in the visual video in the center of the screen (Zhang and Cheng, 2011). For each icon, there were 60 words containing the phoneme ready to be clicked. Trainees may click either icon of phoneme on the screen randomly. After a total of 120 clicks, a progress quiz of 10 words was held. If the accuracy rate was above 90%, the trainee would automatically move on to the next session. If not, they were required to do the training session one more time followed by the quiz. After the repeat session, the trainees could elect to move on to the next level even though the accuracy rate failed to reach 90%.

### Data Collection and Analysis

#### Behavioral Data

In the natural word identification, percent correct accuracy of all tested words (including the trained words and the untrained words) and the untrained words pre-and post-training were compared using repeated measures ANOVA to examine whether there was significant training-induced improvement and generalization. Further simple effect tests and *post hoc* two-tailed t-tests (if needed) were conducted to verify the effects with each group. In analyzing the identification performance for the synthetic continuum, the boundary location, slope and width were calculated with a probit model to fit the individual identification curve (Finney, 1971). The location of the boundary was defined as the point where half the trials of the stimuli were identified as /i/, and the other half was identified as /i/. The boundary width was calculated as the distance between the 25th and 75th percentiles in the fitted identification curve with the probit analysis (Hallé et al., 2004). In order to account for the asymptotic property of the probit model, 0.1 replaced 0% and 99.9 replaced 100% in the individual identification data points. The boundary slope, location and width in the pre- and post- tests were compared between pre- and post- tests using repeated measures ANOVA (group × training) to determine whether training led to significant changes in the identification function. The discrimination data of synthetic continuum was analyzed in terms of percent correct accuracy (Xu et al., 2006). There were four types of comparisons for any given pair of sounds in the form of AB, BA, AA and BB, where stimuli A and B were separated by four steps on the continuum. The step pairs AB and BA included different phonemes while the pairs AA and BB were foil trials with the same phoneme. Discrimination accuracy (P) for each sound pair was determined by the formula of P = P(“S”/S) × P(S)+P(“D”/D) × P(D), with P(“S”/S) representing the percentage of correct “same” responses to the “same” pairs (AA or BB), and P(“D”/D) representing the percentage of correct “different” responses to the “different” pairs (AB or BA).

#### EEG Data

The MMN response was the component of interest in the ERP experiment to measure within- and across- category discriminatory sensitivity. The offline ERP analysis was conducted with BESA (Version 6.1, MEGIS Software GmbH, Germany). The artifacts of the raw data were first corrected to minimize influences of horizontal and vertical eye movements. The auto-correction parameters for horizontal electrooculogram (HEOG) and vertical electrooculogram (VEOG) were 100.0 and 150.0 μV, respectively. Trials with amplitudes beyond ±50 μV were rejected. The bandpass filter was at 0.5–30 Hz, and the data were re-referenced with common average reference. The ERP epoch length was set at 700 ms, including a pre-stimulus baseline of 100 and 600 ms after the onset of the stimulus.

After pre-processing, the MMN responses were derived by subtracting the ERPs for the standard stimulus from the ERPs for the deviant stimuli. Three electrode sites were defined with grouped electrodes in order to improve signal to noise ratio of the data. Left-site electrodes included F3, FC3 and C3, the mid-site electrodes included Fz, FCz, and Cz, and the right-site electrodes included F4, FC4, and C4. The electrode grouping was based on visual inspection of the topographical potential distribution of the MMN responses. A similar approach was used in previous studies (Zhang et al., 2011). The MMN amplitude for each electrode site was quantified by averaging data points within the time window of 40 ms around the MMN peak for each deviant on the individual subject basis. This adaptive window quantification was adopted in consideration of large inter-subject variability in the MMN peak latencies. Individuals’ peaks were looked for 100–300 ms after the stimulus onset. At least 100 accepted deviant trials after artifact correction (120 trials in total) were included in each condition for each subject. For the MMN data collected from the HVPT-E group, repeated measures ANOVA test was conducted to examine effects of training (pre-test vs. post-test), stimulus type (across-category vs. within-category), and electrode site (left, mid, and right). In case of multiple comparisons (e.g., electrode site) in the ANOVA test, the reported *p*-values were Greenhouse-Geisser corrected.

## Results

### Behavioral Results

#### Natural Word Identification

**FIGURE 1:**
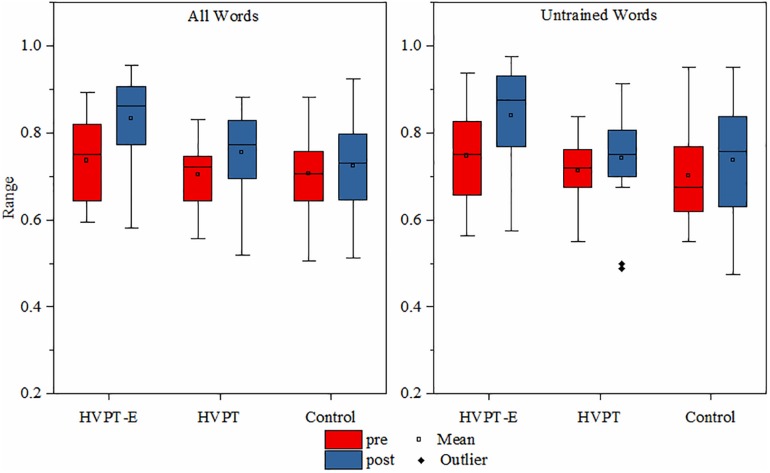
Boxplots for natural word identification of the three subject groups in the pre- and post- tests.

In natural word identification, the percent correct scores of the three participant groups before and after training were compared using repeated measures of ANOVA with training as a within-subject factor and group as a between-subject factor. As expected, there was a significant group effect in the learning outcomes [*F*(2,57) = 5.732, *p* = 0.005, η^2^_p_ = 0.167]. There was also a significant group × training interaction effect [*F*(2,57) = 5.065, *p* = 0.009, η^2^_p_ = 0.151]. Simple effect tests revealed that the identification accuracy of the HVPT-E group significantly improved after training with an increase of 9.7% (*p* < 0.001) (Figure 1). The HVPT group showed a significant improvement in overall natural word identification accuracy as well (*p* = 0.003), with an increase of 5.2%. In contrast, the control group before and after training did not change significantly (*p* = 0.315). Further comparison between the two training groups showed that the HVPT-E group showed greater improvement as reflected in larger progress in natural word identification performance produced by new talkers (*p* = 0.021).

Transfer of learning was examined with untrained word identification (Figure 1). Notably, equivalent amount of gain in the pre- and post- tests was found in the HVPT-E group with an increase of 9.2%. Repeated measures ANOVA showed significant main effects for training [*F*(1,57) = 17.056, *p* < 0.001, η^2^_p_ = 0.230] and group [*F*(2,57) = 3.372, *p* = 0.041, η^2^_p_ = 0.106]. Further comparisons showed significant differences between the HVPT-E group and the HVPT group (*p* = 0.038) as well as between the HVPT-E group and the control group (*p* = 0.022).

#### Identification of the Synthetic /i/-/i/ Continuum

**FIGURE 2:**
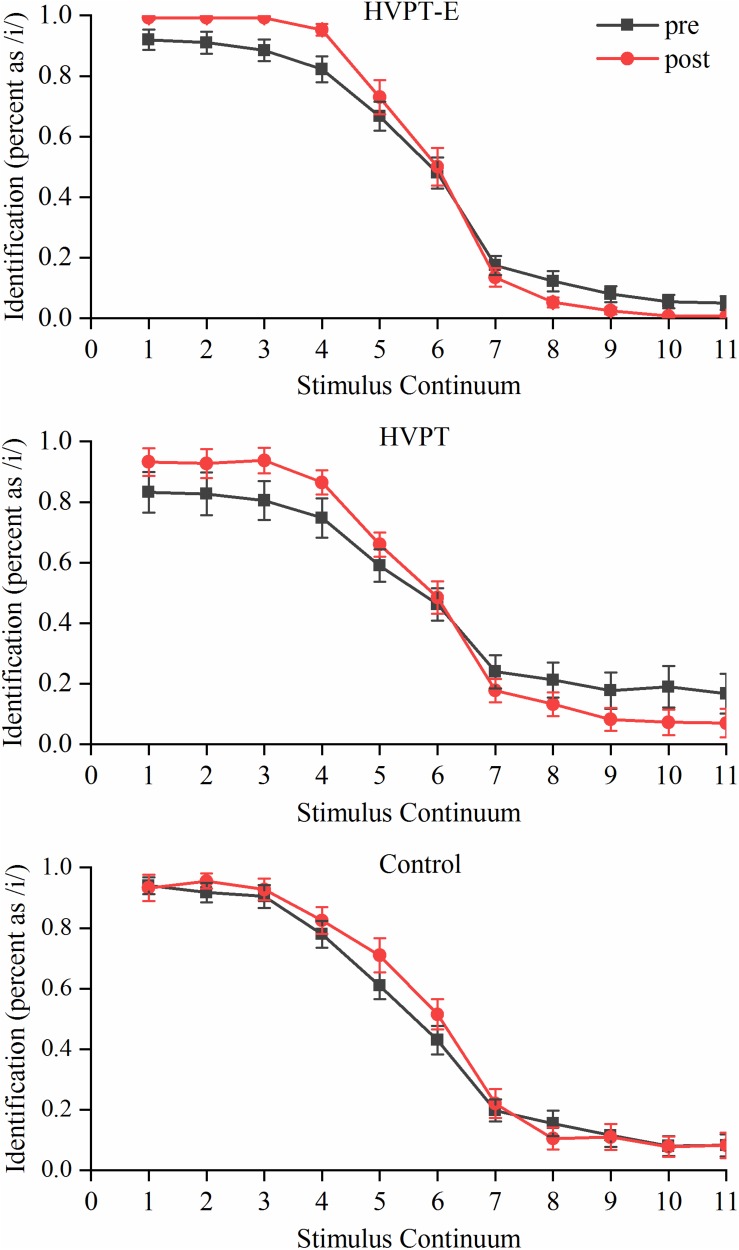
Identification functions of the /i/-/i/ synthetic continuum for the three groups in pre- and post- tests. The data points in the plots represent means + standard error (SE).

Figure 2 depicts the identification performance of the three groups for the /i/-/i/ continuum in the pre- and post- tests. The boundary slope, location and width in the pre- and post- tests of three groups were compared with repeated measures ANOVA test. Significant group × training interaction effects were found in the boundary slope [*F*(2,57) = 6.213, *p* = 0.004, η^2^_p_ = 0.179]. Simple effect test results showed that significantly steeper boundary slopes were observed after training in the HVPT-E group (*p* < 0.001) and the HVPT group (*p* = 0.004), in contrast with no significant changes in the control group (*p* = 0.313). Regarding the boundary width, there was a significant main effect for training [*F*(1,57) = 7.317, *p* = 0.009, η^2^_p_ = 0.114]. But there were no significant changes in the boundary location before and after training. As the synthetic stimuli were controlled for duration, both the HVPT-E and the HVPT group demonstrated training-induced effects on synthetic phoneme identification solely based on spectral cues, as reflected by significantly steeper boundary slopes after training. Notably, there was a significant group effect in the slope change [*F*(2,57) = 7.034, *p* = 0.002, η^2^_p_ = 0.198]. Further comparison between the two training groups showed that the HVPT-E group had larger training-induced improvement than the HVPT group with more robust boundary effect, as indicated by the steeper slope in the identification function (*p* = 0.007).

#### Discrimination of Across- and Within- Category /i/-/i/ Stimuli

**FIGURE 3:**
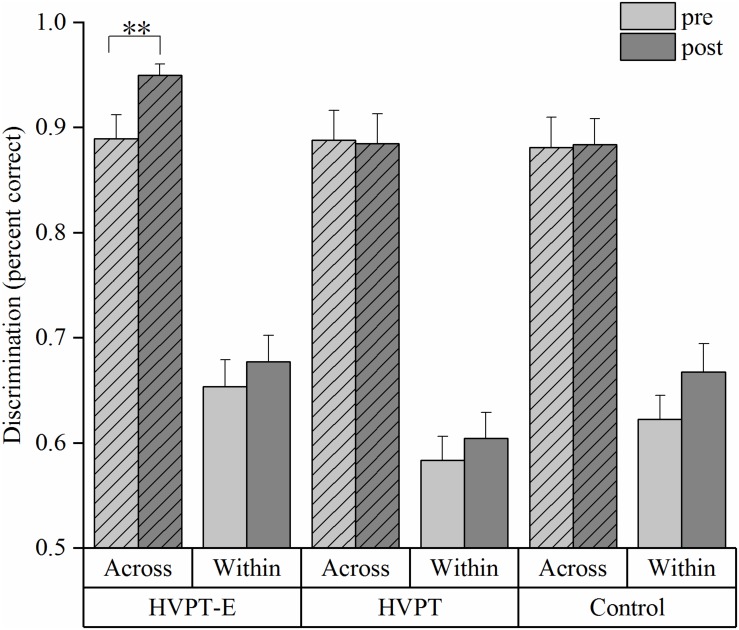
Discrimination accuracy of across- and within- category stimulus pairs by the three groups. The bar plots represent means + standard error (SE) (^∗∗^*p* < 0.01).

The percent correct accuracy data of the across-category pair (Step 3 vs. Step 7) and the within-category pair (Step 7 vs. Step 11) discrimination in the pre- and post- tests were compared using repeated measures ANOVA (group × training) (Figure 3). A significant group × training interaction effect was found in the across-category pair discrimination [*F*(2,57) = 3.973, *p* = 0.024, η^2^_p_ = 0.122]. Simple effect test revealed that only the HVPT-E group had significant improvement after training in discriminating across-category stimuli (*p* = 0.001). In stark contrast, no significant pre-post change was observed in the HVPT group (*p* = 0.861) or the control group (*p* = 0.888). Contrary to our expectation, the within-category pair discrimination data also showed a significant main effect for training [*F*(2,57) = 4.366, *p* = 0.041, η^2^_p_ = 0.071].

### ERP Results From the HVPT-E Group

**FIGURE 4:**
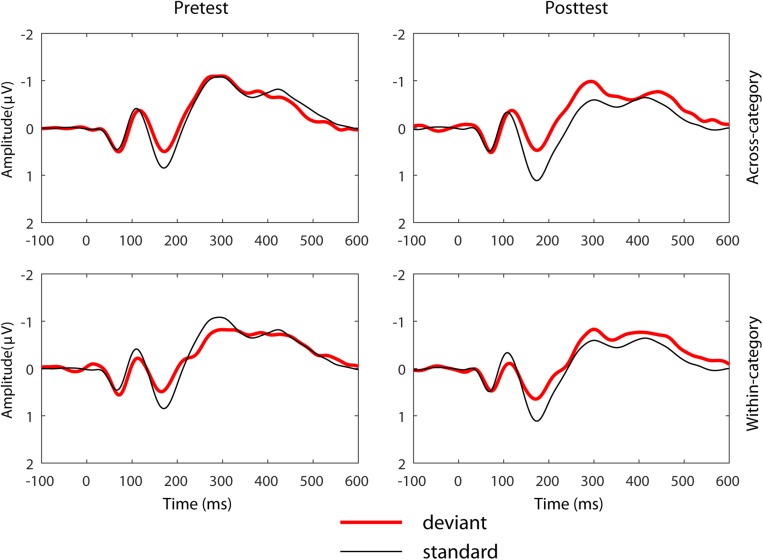
Pre- and post- training grand mean ERP waveform data at the mid-central site for the across- and within- category contrasts for the HVPT-E group.

**FIGURE 5:**
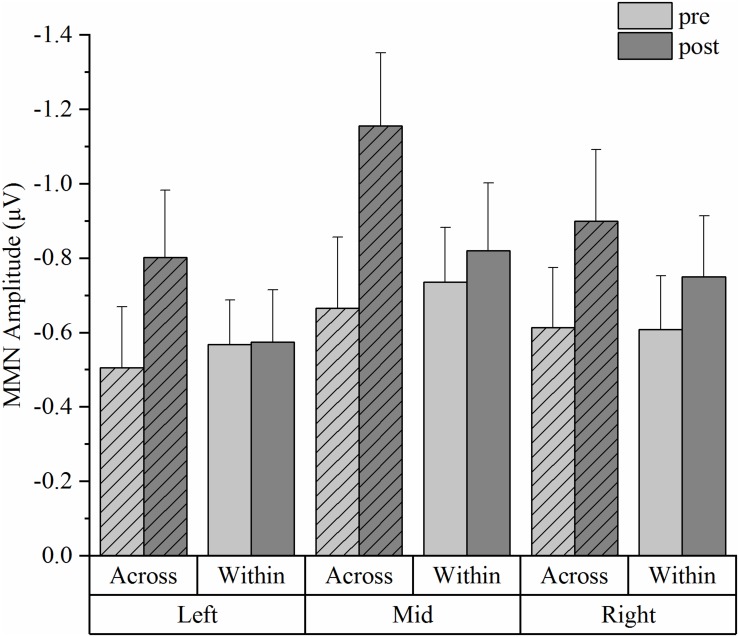
Mismatch negativity (MMN) mean amplitudes from the HVPT-E group for the across- and within- category deviants in pre- and post- tests across the three electrode sites (the left site vs. the mid site vs. the right site). The bars represent means + standard error (SE).

Figure 4 depicts the grand-average ERP waveforms of 20 participants in the HVPT-E group for the across-category deviant, the within-category deviant, and the standard in the pre- and post- tests. In the MMN amplitude data (Figure 5), repeated measures ANOVA revealed significant effects for the main factors of training (pre vs. post) [*F*(1,19) = 5.278, *p* = 0.033, η^2^_p_ = 0.217], deviant type (across vs. within) [*F*(1,19) = 13.447, *p* = 0.002, η^2^_p_ = 0.414], and electrode site (left, mid, right) [*F*(2,38) = 7.826, *p* = 0.002, η^2^_p_ = 0.356].

**FIGURE 6:**
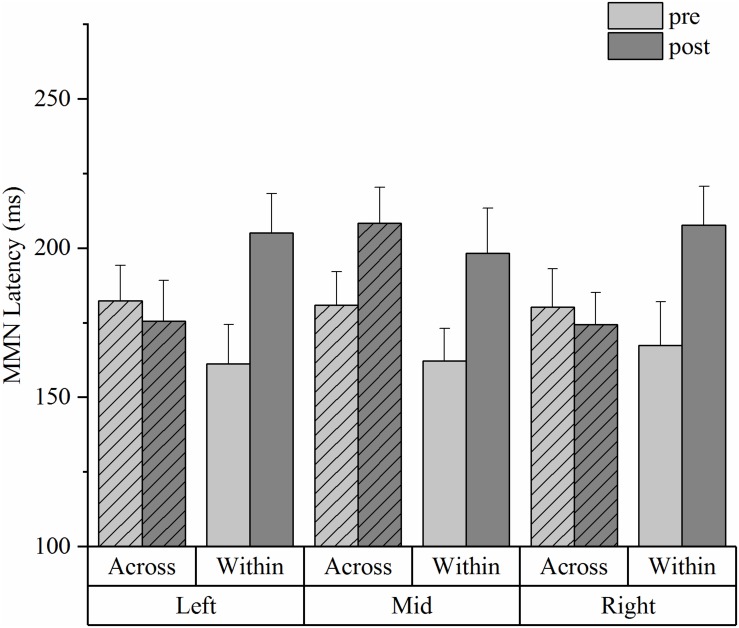
Mismatch negativity mean latencies from the HVPT-E group for the across- and within- category deviants in pre- and post- tests across the three electrode sites (the left site vs. the mid site vs. the right site). The bars represent means + SE.

In the MMN latency data (Figure 6), repeated measures ANOVA showed a significant main effect of training [*F*(1,19) = 4.678, *p* = 0.044, η^2^_p_ = 0.196] and a significant interaction effect of deviant type and training [*F*(1,19) = 5.407, *p* = 0.031, η^2^_p_ = 0.222]. Simple effect tests further showed that the latency of the across-category deviant did not significantly change in the pre-post comparison across the left, mid and right electrode sites (*p* = 0.680). However, the MMN peak latency for the within-category deviant was significantly delayed after training when compared with that of the pre-test [*F*(1,19) = 8.288, *p* = 0.010, η^2^_p_ = 0.198].

## Discussion

The current study tested the role of temporal acoustic exaggeration by comparing the training effects of the modified HVPT paradigm integrating the characteristic of temporal exaggeration in IDS with that of the canonical HVPT design. Forty native Chinese adult learners of English were assigned to two training groups in the self-paced training program that included seven sessions lasting approximately 50 to 90 min, and another 20 subjects served as the control group with no training. Twenty out of the 40 trainees heard naturally-produced words containing the target phonemes (e.g., *sheep* vs. *ship*) by four native American English talkers during the training (the HVPT group) while the other 20 participants heard acoustically-exaggerated words by the same four talkers during the training (the HVPT-E group) with an adaptive training design. The word types, presentation order and frequencies were matched across conditions. We administered pre- and post- tests to assess training effects, including a natural word identification test, synthetic phoneme identification and discrimination tests, and an ERP test for the HVPT-E group. We predicted improvements both in the HVPT-E and HVPT group with greater improvement in the HVPT-E group, given the literature on benefits of acoustic exaggeration and HVPT in enhancing non-native phonetic learning (Logan et al., 1991; Lively et al., 1993; Iverson et al., 2003; Zhang et al., 2009). Consistent with our prediction, the behavioral results showed the advantage of the HVPT design. Both the HVPT group and the HVPT-E group showed significant improvement in naturally spoken words identification produced by new talkers as well as significant changes in synthetic phoneme identification based on spectral cues. Critically, data of the HVPT-E group revealed greater amount of improvement in words identification produced by new talkers and effective generalization to new words (i.e., new phonetic contexts). Further, the HVPT-E group exhibited more native-like CP reflected in both synthetic phoneme identification and discrimination behavior in the utilization of spectral cues. Thus consistent with our previous pilot study (Cheng and Zhang, 2013), the results highlighted the benefit of temporal acoustic exaggeration and adaptive training in training Chinese adults to perceive the English /i/-/i/ vowel contrast.

Consistent with previous research (e.g., Lively et al., 1993), our behavioral data showed that the both training approach (i.e., HVPT and HVPT-E) improved the performance of Chinese adults in perceiving the English /i/-/i/ vowel contrast, which generalized to new talkers. The small amount of gain in the HVPT group suggests that other factors such as learners’ perceptual abilities, L1 backgrounds, and the nature of speech categories to be learned that may limit the efficacy of the HVPT paradigm (Iverson and Evans, 2009; Perrachione et al., 2011; Sadakata and Mcqueen, 2014). For example, Wade et al. (2007) suggested that benefits of HVPT varied across vowel categories, and could disappear for highly confusable vowels. The English /i/-/i/ distinction is reported to be generally hard for a great number of L2 learners regardless of amount of L2 experience (Bohn and Flege, 1997; Flege et al., 1997; Cebrian, 2006; Kondaurova and Francis, 2008; Escudero et al., 2012). Additionally, according to the PAM, the English /i/-/i/ distinction could be particularly hard for Chinese adults because they are both assimilated into a single Mandarin Chinese /i/ category (Best, 1995). Thus, the limited effectiveness of HVPT on Chinese learners’ improvement and generalization is not utterly surprising.

The greater learning effects in the HVPT-E group provided evidence for the need to incorporate the temporal exaggeration in the HVPT paradigm to aid the participants in perceiving the non-native /i/-/i/ contrast. It has been argued that learners’ ability to transfer what they have learned about the contrast distinction to other new stimuli is a result of extracting category-relevant information from training input and developing phonologically constant categorical representations, which can accommodate and be facilitated by a great range of exemplars (e.g., Logan et al., 1991). Developing categorical representations for speech sounds need to involve changing the perceptual weighting of different acoustic cues that contrast the phonetic categories, especially when certain cues critical for native speakers may be weighted as secondary by L2 learners of that language (Bion et al., 2006; Zhang et al., 2009; Ylinen et al., 2010; Giannakopoulou et al., 2013). However, the specific learning mechanism underlying category acquisition may not be well captured by tests with naturally spoken stimuli which have rich redundancy of acoustic cues to indicate phonemic contrasts. For instance, Bion et al. (2006) examined both perception and production of English front vowels by seventeen Brazilian L2 learners. On perception tests with natural speech stimuli, many subjects got high scores comparable to those of native English speakers, whereas these L2 learners’ performance was much lower than the native English speakers in tests with synthetic stimuli that controlled vowel duration length and varied only in spectral cues. It is interesting to note that the pretest results for natural and synthetic stimuli in our study showed a reversed pattern with the relatively higher difficulty with the naturally spoken words. In particular, the literature has well documented that to distinguish the /i/-/i/ contrast, native English speakers rely predominantly on the spectral cues (Mermelstein, 1978; Hillenbrand et al., 2000). In contrast, L2 learners of English, Chinese included, tend to make their judgment based on duration cues (Grenon et al., 2019). Therefore, although the two trained groups showed significant improvement in natural word identification, it is hard to determine whether these trainees learned to use primary spectral cues to identify the target vowels without looking at the evidence from the identification and discrimination data from the duration-controlled synthetic stimuli.

Further evidence on whether the trainees had formed more robust abstract tense and lax categories comes from assessment of the learners’ categorical perception that taps into a fine-grained examination of transfer of learning and training-induced changes in the utilization of the spectral cues. In the CP tests in which only spectral cues were available, both the HVPT group and the HVPT-E group showed significantly steeper slope after training, revealing a more abrupt membership shift between the two categories of /i/-/i/. However, only the HVPT-E group further demonstrated a significant training-induced improvement in the across-category discrimination accompanied by no significant change in the within-category discrimination, indicating enhanced sensitivity at the category boundary in the HVPT-E group. Like the control group, the HVPT group showed no significant pre-post changes in the across- and within- category discrimination data. The group differences in the pre- and post- test results with the synthetic stimuli showed that the HVPT method integrated with temporal acoustic exaggeration succeeded in improving the learners’ ability to attend to and utilize the primary spectral features with more native-like categorical perception of the /i/-/i/ contrast. As duration was strictly controlled in the synthetic stimuli, the significant training effects in the HVPT-E group reflected a true training-induced change in perceptual weighting of the spectral cues that is not attributable to enhanced sensitivity to the secondary duration feature.

Previous studies also reported on changing L2 cue weighting by modified HVPT techniques (Ylinen et al., 2010; Giannakopoulou et al., 2013; Grenon et al., 2019). For example, Ylinen et al. (2010) trained adult Finnish native speakers who relied on duration to identify the /i/-/i/ contrast before training by using the HVPT with modified acoustic stimuli which had equal durations between the vowels. Their training data showed significant improvement in identification scores for both natural and duration-equated stimuli. More recent research by Hu et al. (2016), however, showed that phonetic training does not have to resort to the HVPT method. By controlling secondary cues in the input stimuli, vowel perception training in one single phonetic context produced by one single talker could also significantly change listeners’ perceptual weighting strategy. Specifically, equalizing vowel duration in the training stimuli without HVPT successfully reduced native Chinese listeners’ reliance on the duration cue and improved their use of spectral cues in identifying the English /i/-/i/ vowels. This line of speech training studies supports the experience-driven “attention to dimension” (or “A2D”) models of speech perception (McClelland, 2001; Francis and Nusbaum, 2002; Zhang et al., 2009), which consider perceptual learning as a process of specific changes in attentional distribution by reallocating the learners’ attention on the relevant acoustic dimension which is critical for the L2 phonetic contrasts. For new phonetic categories to be learnt, the perceptual dimensions that are relevant to the category formation should be perceptually “stretched” while irrelevant dimensions should be “shrunk.” Thus the underlying assumption is that the training experience with the absence of duration cues may force the listeners to pay attention to other perceptual cues (e.g., spectral cues) for non-native vowel perception (Ylinen et al., 2010; Giannakopoulou et al., 2013; Hu et al., 2016). In our modified HVPT-E approach, the varying levels of temporal exaggeration for both target vowels provided more variable range of duration than in the natural stimuli, which lead to native Chinese listeners’ increased reliance on spectral cues for English /i-i/ contrast. Although increased variability could be detrimental to efficient discrimination in that as clusters of exemplars increase in size, effective borders of the clusters will shrink or overlap (Eaves et al., 2016), we speculate that the introduction of irrelevant variability along duration dimension could encourage the learners to resort to other more stable cues (i.e., spectral cues) and achieve effects similar to those of the inhibitory training methods (Francis et al., 2000; Holt and Lotto, 2006; Kondaurova and Francis, 2010). For example, Kondaurova and Francis (2010) compared the effectiveness of three training methods (i.e., adaptive training with controlled duration, inhibition training with variable duration, and prototype training) on training native Spanish listeners to perceive English vowel contrast /i/-/i/. They showed that inhibition training was more effective than the other two methods in terms of withdrawing attention from vowel duration. Assuming that reduced reliance on duration cues would make the weight of spectral cues relatively heavier than that of duration cues for perceiving the /i-i/ contrast, it remains to be tested whether the modified HVPT by adding variance along irrelevant dimension could actually reduce learners’ reliance on duration cues, thus helping Chinese learners reach the native-like preference of cue weighting in perceiving English vowel contrast /i/-/i/.

Corroborating evidence from the ERP data in the HVPT-E group demonstrated significant training effects in the stage of pre-attentive cortical processing of the speech sounds, which are reflected by increased MMN amplitude for the across-category deviant after training and increased MMN latency for the within-category deviant after training. The training-induced MMN enhancement for the across-category deviant not only confirmed the behavioral training results of sharpened categorical perception but also demonstrated the important role of acoustic exaggeration in promoting neural plasticity for L2 phonetic category acquisition in adulthood. Our results are consistent with previous research showing enhanced MMN responses for training-induced improvement in speech perception (Kraus et al., 1995; Menning et al., 2002; Zhang et al., 2009). More importantly, the MMN enhancement effect in the pre-post comparison was observed only for detecting across-category differences (Winkler et al., 1999; Sharma and Dorman, 2000; Nenonen et al., 2003), which was accompanied by delayed MMN responses for detecting within-category acoustic differences.

One puzzling phenomenon is that while the post-test MMN results for across-category and within-category contrasts reflect native-like behavioral categorical perception in the HVPT-E group, the pre-test MMN data did not appear to show the same pattern consistent with the behavioral results because the pre-test MMN responses for the across-category contrast did not differ from those for the within-category contrast. According to Ylinen et al. (2006), the MMN component may not necessarily reflect the phoneme boundary effect but index prototypicality of the stimuli. We find this interpretation applicable to our pre-test MMN data. For the Chinese adult subjects, both steps 7 and 11 in the synthetic /i-i/ continuum could be treated as non-prototypical /i/ sounds as the phoneme /i/ does not exist in Mandarin Chinese. By contrast, Step 3 was heard as the /i/ sound, which exists in the Chinese vowel inventory. Thus, the so-called across-category contrast (Step 3 vs. Step 7) prior to training might be treated by the Chinese listeners at the pre-attentive level as acoustic difference between a prototypical vowel and a non-prototypical vowel whereas the within-category contrast (Step 7 and Step 11) might be treated as acoustic difference between two non-prototypical sounds. In this regard, the post-test MMN changes in HVPT-E group could also be viewed as fundamental training-induced changes in evaluating stimulus prototypicality relevant to the L2 phonemic contrast at the pre-attentive level, which would give rise to more native-like categorical perception results (Kronrod et al., 2016).

The MMN results in our study are also in accordance with previous evidence that cue weighting is language-specific in establishing long-term representations of phonetic categories. The enhanced MMN responses on detecting across-category differences in the duration-controlled synthetic vowel stimuli indicate that the training resulted in fundamental changes in cortical representations and automatic processing of the spectral cues that are important for the speech contrast. These categorical representations may experience repeated reinforcement from the training input in the form of attentional reallocation to the critical L2 features to become permanent. This is consistent with the Native Language Neural Commitment (NLNC) theory, which considers phonetic learning as an implicit self-reinforcing computational process. During L1 acquisition, the self-reinforcing process leads to neural commitment with increased sensitivity and efficiency to process the phonological patterns of the native language (Kuhl et al., 2008). This theory also claims that neural commitment can be reversible in adulthood with enriched exposure that can induce substantial plasticity for L2 learning. Improved post-training performance in both HVPT and HVPT-E groups and the MMN responses of the HVPT-E group in our study lend support to the theory by demonstrating substantial neuroplasticity in adulthood, which can be harnessed by proper treatment of the input acoustic properties and delivery mechanism. It is also important to note that there was a large scale of inter-participant variability in the MMN data. According to Kuhl et al. (2008), the degree of plasticity in L2 learning hinges on the stability of the underlying perceptual representations. Short periods of perceptual training in a laboratory setting might not be adequate to affect some participants’ neural structures due to the instability of the phonetic representations for the L2 sounds and interference from their L1 phonetic representations. This calls for more fine-grained research to probe the differences in individual learners in a longitudinal design.

The behavioral data together with the MMN results provided strong evidence for the method of HVPT integrating temporal exaggeration to aid the learners in forming more native-like categories of the English /i/-/i/ contrast, which has been shown in previous research (e.g., Zhang et al., 2009). However, the previous studies did not specifically separate the relative contributions of acoustic exaggeration and HVPT. To our knowledge, this is the first study to highlight the specific role of acoustic exaggeration in improving participants’ perception of the /i/-/i/ contrast, particularly in terms of categorical perception based on the critical spectral cues with or without attentional focus. Research has shown that acoustic exaggeration may enhance the cues distinguishing the linguistic features of the native language from very early in infancy (Ratner and Luberoff, 1984; Kuhl et al., 1997; Liu et al., 2003; Zhang et al., 2011). In adults, Uther et al. (2012) showed hyperarticulation of vowels elicited larger MMN response in both native and non-native speakers of English, suggesting that acoustic exaggeration could increase neural sensitivity to speech contrasts in second language learners, which may facilitate phonetic learning. Our study confirmed that acoustic exaggeration can be incorporated in training materials to help non-native adult learners establish more robust abstract sound categories along relevant dimensions. Additionally, it is worth noting that in contrast to the previous training studies mostly lasting long hours or even weeks (Bradlow and Pisoni, 1997; Callan et al., 2003; Zhang et al., 2009), the modified HVPT-E protocol required much less time to achieve equivalent amounts of gain. Presumably, the greater acoustic variety and characteristic details can facilitate the formation of prototypical representations of the phonetic category in both L1 and L2 acquisition (Kuhl et al., 2008). But it remains to be tested whether such L2 training effects are sustainable and transferable from perception to production in the long term. It is also unknown how generalizable the HVPT approach with acoustic exaggeration is for different types of L2 vowel and consonant contrasts and whether it is applicable to clinical populations such as those with severe hearing loss (Miller et al., 2016).

One inherent confound to the interpretation of the observed advantage in the HVPT-E group in our study is that temporal acoustic exaggeration provided more exposure time to the vowels as the HVPT-E stimuli were 200% longer than the HVPT stimuli in the beginning of the training program. However, it is noteworthy that according to the training records, the total training time for an average participant in the HVPT-E group was approximately 10 min more than the average person in the HVPT group. Given the L2 phonetic training literature, it seems unlikely that an extra 10 min of training itself would result in significant differences between the HVPT-E group and HVPT group. Further study could be designed to verify this speculative statement here by controlling the stimulus exposure time in the training sessions. A second limitation is that as the ERP data in the current study were only from the HVPT-E group, a full examination of the group differences in the ERP data could not be conducted to strengthen the findings at the neurophysiological level. A third important limitation of the current study is that unlike our previous work (Cheng and Zhang, 2013) with participants who did not major in English, the participants in the current report were all studying English in a highly selective university in China. They appeared to show relatively high (near-native) level of categorical perception before training, as indicated by much higher across-category discrimination accuracy compared to within-category discrimination in all three groups. Future studies can be conducted to include a wider range of individual L2 proficiency to determine the generalizability and effectiveness of the HVPT-E approach.

## Summary

This study provided direct evidence that high variability phonetic training with temporal acoustic exaggeration was more effective than the canonical HVPT. Both behavioral and electrophysiological data indicated significant training and generalization effects of the temporally-exaggerated HVPT on the Chinese ESL learners’ perception of English /i/-/i/ contrast. The results demonstrate great plasticity of non-native phonetic learning in adulthood induced by enriched input in a software training program, which has important implications for second language pedagogy as well as theories on language learning.

## Ethics Statement

This study was approved from the Institutional Review Board for Biomedical Research at Xi’an Jiaotong University (IRB Code Number: 2018-553). Written informed consent was obtained from each participant.

## Author Contributions

BC and YZ conceived the study. BC, XZ, and YZ wrote the manuscript. All authors designed the study, collected and analyzed the data, read and approved the manuscript and agree to be accountable for all aspects of the work.

## Conflict of Interest Statement

The authors declare that the research was conducted in the absence of any commercial or financial relationships that could be construed as a potential conflict of interest.
